# Intraosseous Hemangioma of the Zygomatic Bone: A Rare Maxillofacial Case in a 57-Year-Old Woman

**DOI:** 10.1155/crid/4813808

**Published:** 2025-09-01

**Authors:** Bahareh Hekmat, Farhad Aghmasheh

**Affiliations:** Department of Oral and Maxillofacial Radiology, School of Dentistry, Zanjan University of Medical Sciences, Zanjan, Zanjan Province, Iran

**Keywords:** bone, hemangioma, zygomatic

## Abstract

Central hemangioma is one of the rare lesions of the jawbones, with a prevalence ranging between 0.5% and 1%. It more commonly occurs in the vertebral column and cranial bones, with rare occurrences in the jaws. The World Health Organization classifies hemangioma as a true neoplasm of endothelial origin; however, some authors believe that hemangiomas are hamartomas. Diagnosis of this lesion can be challenging due to its varying clinical and radiographic characteristics. Treatment is also difficult due to the presence of an extensive vascular network in the affected area. In this article, we present a case of central hemangioma in the body of the zygomatic bone of a 57-year-old female, which was treated through surgical resection.

## 1. Introduction

Hemangioma is a benign neoplasm of endothelial origin, characterized by the proliferation of blood vessels, forming a mass similar to a neoplasm, although in many cases it is considered a hamartoma [1, 2]. Hemangiomas can occur in any part of the body but are most commonly seen on the skin and subcutaneous tissues [3, 4].

Mandibular hemangiomas are twice as common as maxillary ones, with the most affected areas in the mandible being the posterior body, ramus, and the inferior alveolar canal. Some tumors can also involve the condyle. Hemangiomas typically appear as multilocular, radiolucent lesions with well-defined cortical borders, though in some cases, the lesions may have ill-defined margins, mimicking a malignant appearance [5, 6].

Hemangiomas are more common in females with a 2:1 ratio. The tumor frequently develops during the first and second decades of life, but it can also occur later in life. It is often asymptomatic, though symptomatic cases do exist. The lesion grows slowly, and the resulting swelling may or may not be painful. It is usually nontender and has a “bony hard” consistency. When pain is present, it is typically throbbing in nature. The lesion may cause elastic mobility (rebound mobility) and migration of the teeth in the affected area. Gingival bleeding around the crowns of the involved teeth may also occur [7–9].

Panoramic radiographs, CT scans, and MRI are the imaging modalities commonly used for diagnosing this lesion [10].

This article presents a rare case of central hemangioma occurring in the body of the zygomatic bone.

## 2. Case Presentation

A 57-year-old woman presented to an oral and maxillofacial radiology center with a complaint of a firm, painless swelling along the lower margin of the right orbit. She was aware of a previous lesion and had undergone surgery in the same area 2 years prior. Clinical examination revealed a bony-hard, painless swelling on the right cheek. There were no limitations in eye movement, and the skin over the lesion appeared normal. Examination of the temporomandibular joint (TMJ) showed no abnormalities. The patient has no significant medical history, does not take any specific medications, has no history of alcohol or tobacco use, and reports no prior trauma in the region of interest.

**Figure 1:**
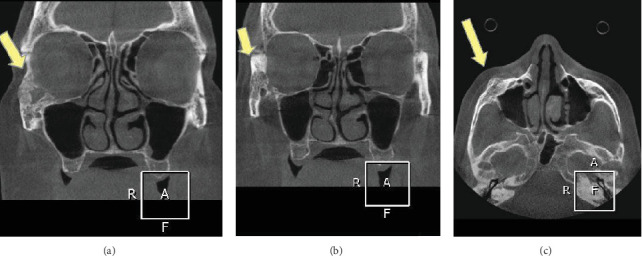
(a, b) Coronal CBCT scan and (c) axial CBCT scan of central hemangioma in the zygomatic bone.

A CBCT scan revealed a heterogeneous, expansile lesion with ill-defined (blending) margins in the right zygomatic bone, extending to the outer half of the inferior border and the lateral wall of the orbit. Hyperdense areas appeared amorphous. Expansion of the zygomatic bone and soft tissue of the cheek was observed (Figure 1).

Based on the radiographic appearance, differential diagnoses included osteoma (trabecular), osteoblastoma, and cemento-ossifying fibroma.

The surgical procedure was performed under general anesthesia with nasoendotracheal intubation. Preoperative imaging was used to define the extent of the lesion and its relation to adjacent anatomical structures. Following standard aseptic preparation and draping, a hemicoronal incision was selected based on lesion size, location, and aesthetic considerations. Subperiosteal dissection was carried out to expose the lateral midface and zygomatic arch.

The lesion was carefully delineated, and en bloc resection of the involved bony segment was performed with adequate safety margins to ensure complete removal and reduce the risk of recurrence. Hemostasis was meticulously maintained throughout the procedure, often requiring bipolar cautery or hemostatic agents due to the vascular nature of the lesion.

Reconstruction of the resulting defect was achieved using autogenous bone grafts and titanium mesh. The soft tissues were repositioned and sutured in layers. The patient was monitored postoperatively for signs of bleeding, infection, and functional and aesthetic complications. Follow-up imaging was scheduled to confirm complete excision and assess for potential recurrence. The timeline of clinical events is summarized in Table 1.

Following surgical excision of the intraosseous lesion, histopathological evaluation under light microscopy revealed a benign vascular lesion composed of numerous small, thin-walled capillary vessels. These vascular channels are irregularly arranged in a lobular pattern within a fibrous stroma. The vessels are lined by a single layer of bland endothelial cells, showing no cytologic atypia or mitotic activity.

**Figure 2:**
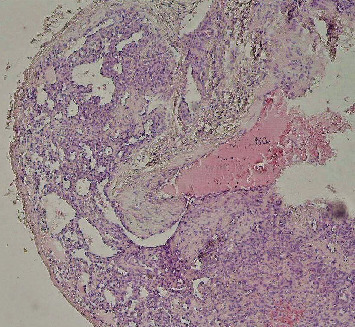
Photomicrograph of intraosseous capillary hemangioma showing numerous small, thin-walled vascular channels lined by bland endothelial cells within a fibrous stroma (H&E stain, original magnification ×100).

In part of the sample, intertrabecular bone spicules are observed adjacent to the lesion, supporting its intraosseous location. No evidence of active hemorrhage, thrombosis, or necrosis is noted in the examined section. There are no features suggestive of malignancy or aggressive behavior. These histological features are consistent with a diagnosis of intraosseous capillary hemangioma (Figure 2).

## 3. Discussion

Central hemangioma is a rare benign neoplasm of endothelial origin. Lesions can present as painless swellings that may cause mild to severe facial asymmetry. Pain and paresthesia are not characteristic features of this condition [11, 12].

Various radiographic appearances have been described by authors for this lesion. It is most commonly described as multilocular, with small radiolucent cavities that resemble enlarged marrow spaces, surrounded by thick, dense, and well-defined trabeculae. These trabeculae create a honeycomb appearance of small, round radiolucent spaces, indicative of blood vessels aligned with the x-ray beam. Radiopaque presentations in hemangiomas are rare. A classic “sun-ray” pattern has been proposed by different authors, representing thickened bone trabeculae oriented perpendicular to the bone surface [7, 12, 13].

The differential diagnosis includes multilocular lesions such as ameloblastoma, central giant cell granuloma, myxoma, and aneurysmal bone cyst [2]. Hemangiomas are classified histologically into three types: capillary, cavernous, and mixed. The proliferating endothelial cells create a network of vascular spaces. Dhiman et al. and Brad et al. described the developmental stages of hemangiomas in three phases: early (highly vascular), intermediate (exhibiting blood clotting), and terminal (various stages of ossification) [10, 14].

Different therapeutic modalities are suggested for treating hemangiomas, with the choice depending on lesion size, patient age, and potential complications of treatment. These treatments include surgery, radiotherapy, sclerosing agents such as sodium morrhuate or psylliate (which are injected into the lesion), and cryotherapy [12]. In more problematic or life-threatening hemangiomas, medical treatment may be applied. In some cases, corticosteroids are effective in reducing the lesion size, with a response rate of 70%–90%. Ligation of the external carotid artery effectively reduces the risk of hemorrhage during surgery and is considered the best treatment option for hemangiomas [14, 15]. Hemangiomas exhibit various clinical and radiographic presentations, and their misdiagnosis or improper treatment can lead to fatal outcomes. Treatment of this lesion should be carefully planned based on the patient's age, the extent of the lesion, and the patient's medical condition. Some studies suggest that resection of the tumor along with the surrounding normal tissues reduces the risk of hemorrhage and significantly decreases the likelihood of recurrence [3].

## Figures and Tables

**Table 1:** Timeline of clinical events.

**Date/timeframe**	**Clinical event**
~2 years prior to visit	First surgery performed on the right zygomatic region for a similar lesion
1 month	Patient presents with firm, painless swelling on the right cheek
1 week	CBCT imaging reveals expansile intraosseous lesion in the right zygomatic bone
2 weeks	Surgical resection of the lesion under general anesthesia
Post-op Days 0–2	Postoperative monitoring for bleeding, infection, and complications
Post-op Weeks 1–2	Histopathology confirms diagnosis of intraosseous cavernous hemangioma
Post-op Month 1	Follow-up imaging confirms successful resection and no signs of recurrence

## Data Availability

The data will be shared upon reasonable request by the corresponding author.
